# How does the problem-oriented innovation system (PIS) help in the management of cardiovascular diseases?

**DOI:** 10.3389/fpubh.2024.1362716

**Published:** 2024-03-26

**Authors:** Shohreh Nasri, Javad Amani, Gelayol Safavi, Sepehr Ghazinoory

**Affiliations:** ^1^Department of Science & Research Policy, National Research Institute for Science Policy, Tehran, Iran; ^2^Department of Information Technology Management, Tarbiat Modares University, Tehran, Iran

**Keywords:** innovation system, problem-oriented innovation system, health policy, cardiovascular diseases, grand challenge, mission-oriented innovation policy

## Abstract

**Introduction:**

Cardiovascular diseases are a multifaceted and complex problem in the health system that can change the priorities of the economic, social, and even political systems of countries. Therefore, as a grand challenge (GC), its management requires adopting a systematic, interdisciplinary, and innovative approach. In Iran, the most common causes of death, have changed from infectious and diarrheal diseases to cardiovascular diseases since 1960.

**Methods:**

In this study, the novel framework of the problem-oriented innovation system (PIS) has been used, and cardiovascular diseases in Iran have been selected as a case study. To this end, first, the main challenges related to cardiovascular diseases in Iran were identified in two layers of “governance-centered” (including legal and policy gaps, insufficient education, financing, lack and unbalanced distribution of medical personnel) and “society driven” (including unhealthy diet and lifestyle, uncontrolled and hard-to-regulate factors, and high costs) through a library research. Then, the functional-structural framework of the problem-oriented innovation system was used to analyze cardiovascular diseases and provide policy recommendations.

**Results:**

The findings indicate that based on the eight functions of the problem-oriented innovation system, an important part of cardiovascular diseases can be managed and controlled in three short-term, medium-term, and long-term periods.

**Conclusion:**

Increasing public awareness in the form of university courses, participation of the government with the private sector in building and equipping specialized cardiovascular centers, creating an electronic health record from birth, implementing a family health plan focusing on less developed areas, supporting agriculture and guaranteeing the purchase of agricultural products and healthy food, increasing the capacity of accepting students in medical and paramedical fields, and allocating pharmaceutical currency in the form of pharmaceutical subsidies directly to cardiovascular patients, are among the most important policy recommendations for this grand challenge.

## 1 Introduction

Cardiovascular diseases are one of the most common causes of death in the world, which have brought serious challenges to the health systems of countries. In the 2017, Global Burden of Disease study, it was estimated that 17.8 million deaths occurred due to these diseases (1, 2). In 2019, cardiovascular diseases were the underlying cause of 9.6 million deaths in men and 8.9 million deaths in women, which was almost a third of all deaths in the world (3) and it is expected this number will increase to 23.6 million by 2030 (4). Therefore, we are facing a growing problem in most regions of the world (5).

The grand challenge of cardiovascular diseases does not include only the technical and specialized aspect -which originates from the knowledge of the healthcare staff- but also includes the economic, cultural, and political dimensions. However, the fragmented studies have not shown the big picture of the causes of cardiovascular diseases (6). In the realm of such a challenge, there are numerous actors and stakeholders who each pursue different and sometimes conflicting interests. Establishing a relative trade-off between these scattered actors creates complex relationships that may make the problem malignant until solving one aspect of it worsens the other aspects. Therefore, the problem of cardiovascular disease has dimensions and complications that can make its situation chaotic. Therefore, it cannot be expected that effective answers will be found through an institution with single-agent (islanded) policies. Therefore, the innovative response to the grand challenge of cardiovascular diseases requires that first all the extensive relationships and interactions related to the problem are defined in a network of actors and agents that do not have a clear geographical boundary, then, in a systematic attitude, the problem’s aspects are determined and the institutions related to it are also specified or if necessary, established. Additionally, the existing frameworks have not been able to answer the grand challenges. Therefore, a systematic innovation approach called “the problem-oriented innovation system” in innovation studies is applied to solve the problem.

In Iran, the most common causes of death have changed from infectious and diarrheal diseases to cardiovascular diseases since 1960 (7). According to a study conducted about a decade ago, cardiovascular diseases account for about 39.3% of all deaths (8) and according to a 2019 study, they account for about 46% of all deaths and 20 to 23% of the disease burden in Iran (7). Although Iran has taken important steps to control and prevent cardiovascular diseases (9), compared to the countries of the Eastern Mediterranean region, it probably has a higher burden of cardiovascular diseases (10). There is also evidence that the incidence of coronary heart diseases (11) and cardiovascular diseases are increasing in Iran (9). On the other hand, Iran is facing an increasing trend with the increase of its older adult population (12) and with increasing age, death due to cardiovascular diseases increases (13). Considering the need to reduce premature death by 25% from non-communicable diseases by 2025, the Iranian government has been tasked with creating a coordinated national strategy to reduce the burden of cardiovascular diseases (14). Therefore, it is necessary to make policies around this issue under a systematic framework.

Based on the above, in this study, the framework of the problem-oriented innovation system (15) is used to find solutions to manage the grand challenge of cardiovascular diseases in Iran. The main question is: how is it possible to manage the grand challenge of cardiovascular diseases and minimize its adverse economic, social, and cultural effects in society through the problem-oriented innovation system approach? Therefore, we seek to provide policy recommendations to improve the performance of the mentioned innovation system with the structural-functional analysis of the innovation system based on the problem of cardiovascular diseases in Iran. It is worth mentioning that although the case studied is Iran, this systematic framework is expected to be a guide in other countries as well.

To answer the research question, in the second part of the study, after explaining the problem, the challenges of cardiovascular disease extraction, classification and analysis, and the innovative system approaches in solving the problem are explained. In the third part, the research methodology will be presented and then the innovation system framework for cardiovascular diseases in Iran together with its eight functions will be analyzed, and finally, the research results, implications, and policy recommendations will be presented.

## 2 Literature review

### 2.1 The issue of cardiovascular diseases and its dimensions

The knowledge required in dealing with the problem of cardiovascular diseases can be mainly classified into two parts: “professional knowledge” related to diseases and “management knowledge” to control them. Professional knowledge, generally, depends on the knowledge of the medical staff, but management knowledge is of policy, decision, and planning, which requires the thoughts of policymakers. Therefore, the problem finds an optimal answer when the results of these two types of knowledge are used. However, since the actors of both are numerous and the interactions between them are complex and sometimes conflicting, solving the problem and providing solutions and policy recommendations requires a systematic and innovative approach.

Governments have been able to manage part of the challenges of cardiovascular diseases by formulating policies; but most of these policies are scattered and *ad hoc* and have not followed a comprehensive, interdisciplinary, and systematic approach to dealing with the problem. For example, in South Africa, to reduce the increasing rate of cardiovascular diseases, the government formulated policies to reduce salt consumption in the society, which reduced cardiovascular diseases by 11% per year and reduced health costs. It reduced healthcare costs for thousands of families, mostly in middle-income groups (16). In Vietnam, the mass media have taken effective measures to reduce salt and tobacco consumption, with the help of blood pressure or cholesterol-lowering drugs and combinational drug therapy for people at risk (17). In Mexico, regulations related to reducing the content of trans fatty acids in foods have been approved (18). In Iran, due to the significant population of government employees, a program was set up to train government employees in the field of prevention and control of risk factors of non-communicable diseases, especially cardiovascular diseases (8). Countries have followed different approaches in adopting problem management policies: legislative and deterrent approaches, encouraging and supportive policies, educational and awareness-raising recommendations, and a combination of approaches over a period. However, it can be said that most of them have not experienced a comprehensive, systematic, and generalizable framework.

The literature review indicates that the challenges that have led to the spread of cardiovascular diseases can be classified into two main categories. The first category is the “state-oriented” challenges that the main responsibility of solving or facilitating them lies with the government and the parliament, or the state in general. The second category is “society-oriented” challenges, which are created, facilitated, or solved by individuals and society in general. Therefore, in addition to the professional and technical dimensions, the management of the big challenges of cardiovascular diseases, also includes various economic, social, and cultural dimensions. The extracted challenges from the literature were coded, categorized, and listed in Table 1.

**Table 1:** The most important “state-oriented” and “society-oriented” challenges for cardiovascular diseases in Iran.

Challenges		References
legislative and policy gaps (G1)	The infancy of health system policy the complexity of policies and the extent of actors amending, approving, and compiling laws, guidelines, and licenses	Gholipor et al. (19), Mosadeghrad and Rahimi-Tabar (20), Damari et al. (21)
Insufficient training (G2)	Education through the formal system education and training informal education	Kazemi et al. (22), Ghasemi Ghale Ghasemi et al. (23), Shahbazi et al. (24), Rezapour Gatabi et al. (25)
Financing (G3)	Economic burden and high costs weakness of insurance coverage	Emamgholipour et al. (26), Zanjanian et al. (27), Raghfar et al. (28)
Scarcity and unbalanced distribution of treatment staff and medical equipment (G4)	Lack of doctors and medical staff reluctance to enter the residency course in general practitioners unbalanced specialty and geographical distribution of doctors Immigration of doctors and medical staff unbalanced distribution of medical equipment and technologies	Azizi et al. (29), Noori Hekmat et al. (30), Bagheri Lankarani et al. (31), Kazemi (32), Vedadhir and Eshraghi (33), Haji Aghajani et al. (34)
Nutrition and unhealthy lifestyle (P1)	Unhealthy nutrition Incorrect behavior patterns type and lifestyle changes low level of health awareness and literacy	Samavat et al. (8), Darafshi Ghahroudi et al. (35), Shahbazi et al. (24), Vahdani et al. (36), Javadi and Pour Jebeli (37), Pourreza et al. (38), Sadeghi et al. (39), Rezapour Gatabi et al. (25), Kazemi et al. (22), Marzangi et al. (40)
Uncontrollable and hard to adjust factors (P2)	Genetic and racial factors overweight and obesity background, mental, and related diseases	Samavat et al. (8), Shahsavari et al. (41), Sadeghi et al. (39), Javadi and Pour Jebeli (37), Rafieipour et al. (42)
High costs (P3)	High diagnostic, medicinal, and treatment costs	Zanjanian et al. (27), Emamgholipour et al. (26)

The symbols used in the table (G_i_’s and P_i_’s) are used in the following part of the research.

In this study, the state-oriented and society-oriented grand challenges are also categorized based on the “geography,” “nature,” “norm,” and “period of change”:

- The geographical dimension: The problem’s challenges impact on three levels: “regional,” “national” and “international.”
- The nature of the challenges is classified into two aspects: “quantitative” and “qualitative.” Quantitatively, it deals with the factors that can be counted and measured, and how to value them, and qualitatively, it deals with the non-numerical components that are rooted in thought, beliefs, People’s values and perceptions.
- The type of influence and control: This refers to the norms; If the change is based on law, rule, and instruction, it is “legislative norm” and external. If it is based on awareness and subjective knowledge, it is “cultural” and internal.
- The time period of change: this can be “short-term,” “medium-term” or “long-term” depending on the period of the policies’ effect.

**Figure 1:**
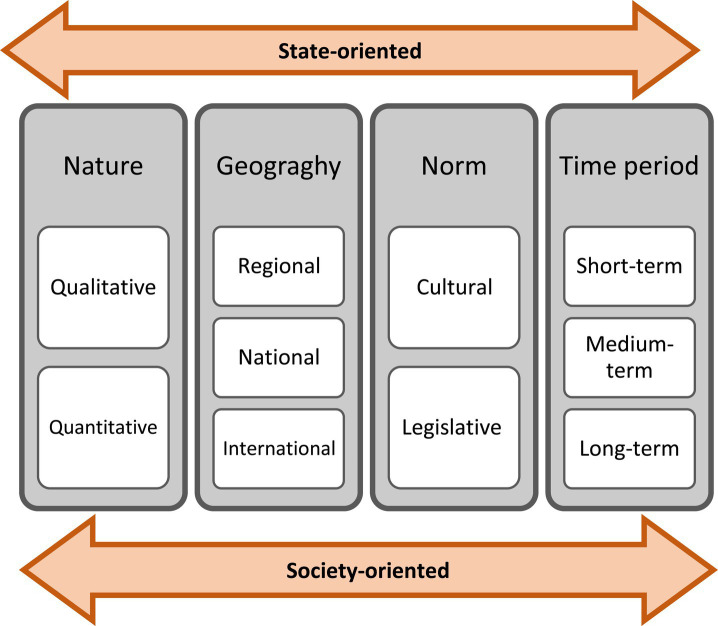
Effective indicators in the management of grand challenges of cardiovascular diseases.

Figure 1 shows the indicators affecting these dimensions in general.

Based on the aforementioned, the relationships between the challenges and indicators related to the grand challenge of heart disease have been analyzed in Table 2. The noteworthy point is that although the indicators are not practically inseparable, this separation is necessary to determine the type of treatment with each factor. For example, the geography of a challenge may be defined at the national level, but in some cases, effective measures have been taken at the regional level. Cultural norms are related to customs and unwritten rules and mainly have an internal control center, but legislative norms mainly have an external control center and are subject to written rules. Another important point is that, solving a challenge cannot necessarily help in solving the problem along with other challenges; In other words, policy instructions and recommendations to solve a challenge in the state-oriented layer may worsen the situation of another challenge in the same layer and even the society-oriented layer. For example, the government may follow the policy of staff retention by increasing salaries, tariffs and financial benefits in order to solve the problem of shortage and uneven distribution of medical personnel. This itself causes the worsening of the financing challenge at the state-oriented level, and if it is provided and compensated through increasing the rate of doctors’ visits, it will make the cost challenge at the society-oriented level even more pathetic. In this way, the cardiovascular disease is a serious problem, so its solution requires a comprehensive approach that systematically classifies and explains the challenges. Also, common methods have not been effective enough and it is necessary to analyze the problem with innovative methods.

**Table 2:** Characteristics of the “state-oriented” and “society-oriented” grand challenges in the problem of cardiovascular diseases.

	Indicator challenge	Geography	Nature	Norm	Time period
State-oriented challenges	Legislative and policy gaps (G1)	National	Quntitative	Legislative	Short-term
	Insufficient training (G2)	National	Qualitative	Cultural	Long-term
	Financing (G3)	National	Quntitative	Legislative	Medium-term / Long-term
	Scarcity and unbalanced distribution of treatment staff and medical equipment (G4)	Regional	Quntitative	Legislative	Long-term
Society-oriented challenges	Nutrition and unhealthy lifestyle (P1)	International	Qualitative	Cultural	Long-term
	Uncontrollable and hard to adjust factors (P2)	International	Qualitative	Cultural	Long-term
	High costs (P3)	National	Quntitative	Legislative	Long-term

### 2.2 Systematic approaches to innovation and response to grand challenges

Different definitions of innovation have been presented, most of which are rooted in Schumpeter’s view (43), which defined it as providing a new product, using a new method, opening a new market, using a new source, and conducting a new organization. But today, innovation has taken on a different meaning and has gone beyond the initial theories and models that saw the innovation process as linear and considered it as a direct product of technical research. Nelson and Winter (44) had a new attitude toward innovation, which put an end to linear approaches to innovation and then became the basis of systematic ideas toward innovation. In the first conceptualization of systematic approaches to innovation, the national innovation system (45) and then other approaches such as the technological innovation system (46), the regional innovation system (47), and finally, the sector innovation system (48) were introduced. Although these approaches offer systematic insights by emphasizing on different cores such as the country, geography, industry and technology, they have shortcomings in the analysis of macro social systems issues. This led to the appearance of the other types of innovation systems. For example, by criticizing the existing approaches of the innovation system on national and sub-national borders, the global innovation system was introduced, which, by focusing on multiple scales, theorizes the development of innovation processes at the global level regardless of geographical boundaries and emphasizes the role of open innovation (49).

Another shortcoming relates to the lack of study on the analysis unit of “macro problem/grand challenge.” The concept of grand challenges refers to national or global societal problems such as energy, environment and health (50). In response to this research gap, social innovation system (51) was introduced with the aim of responding to social challenges through the development of innovation and the mission-oriented innovation systems (52) was introduced with the goal of defining, pursuing and completing a grand challenge or social mission. The problem-oriented innovation system (15) is another approach that explains the gap of not paying attention to macro-level technical and societal problems and challenges, and also raises the question that “How can macro-level technical and societal problems be analyzed through the simultaneous development of social and technological innovations?”

The problem-oriented innovation system is a suitable framework to answer the problem of cardiovascular diseases because it emphasizes the application of knowledge and technological innovations, covers various economic and social dimensions, operates beyond geographical and regional boundaries, considers the simultaneous application of social and technical innovations, and emphasizes on the interaction between actors that may arise from different technological, sectoral and social systems (15). Additionally, various researchers have used this approach in their studies to analyze major challenges such as air pollution (15), water scarcity (53), peace engineering (54), digital divide (55), waste management (56), and Covid-19 (57).

Therefore, since previous studies have rarely considered the problem of cardiovascular diseases through the lens of the innovation system (as a grand challenge whose effective management requires a “systematic” and “multifaceted” approach), this paper analyzes cardiovascular diseases in Iran based on the PIS framework.

## 3 Methodology

The current research is a combination of two methods (descriptive and analytical) using document study. Documents and library resources are used to examine the current state of cardiovascular diseases at the national and global levels and to identify grand challenges through document content analysis and the coding process. In this regard, in the first stage, documents related to cardiovascular diseases were examined. After dividing relevant sentences, theoretical saturation was achieved in extracting codes and concepts.

**Figure 2:**
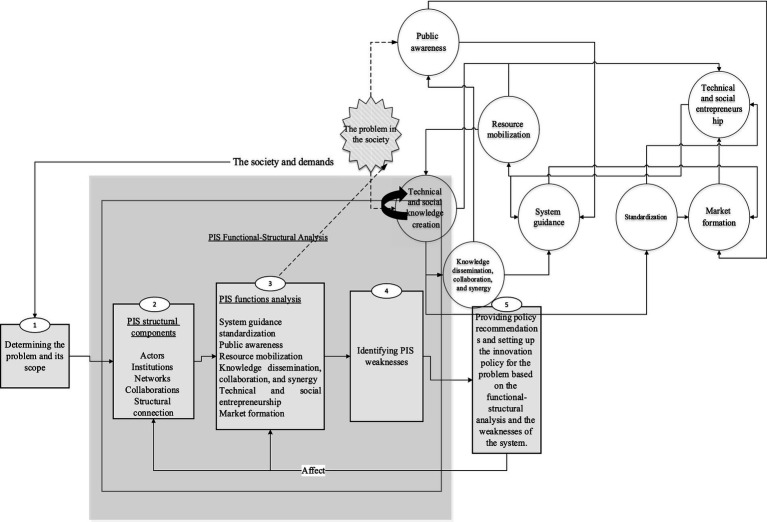
The analytical framework of the problem-oriented innovation system, adapted from Ghazinoory et al. (15).

In the following, according to Figure 2, we will use the analytical framework of the problem-oriented innovation system for the grand challenge of cardiovascular diseases in Iran. PIS is a suitable framework for analyzing issues that require the simultaneous application of technological and social innovations with an interdisciplinary approach (15). Cardiovascular diseases have such characteristics. They are intersectoral and in addition to technological innovations, social innovations play a significant role in dealing with them.

Based on the PIS framework, the issue of the society is first determined based on the needs of the society and the existing demand, and then it becomes a policy priority supported by the government. After that, the structural components of the system which are the operational parts or its actors should be identified and introduced. In the next step, system weaknesses in PIS are identified based on structural components and system functions. Weaknesses are marked with the symbol W and strengths are marked with the symbol S. Finally, based on the conclusions obtained from the previous stages, policy recommendations are presented in order to improve the performance of the innovation system based on the problem of cardiovascular diseases.

### 3.1 Identifying the actors and institutions affecting the issue of cardiovascular diseases in Iran

In order to succeed in systematic problem solving, strong interactions and cooperation between all involved actors and stakeholders should be established to enable the multidimensional aspects of the problem-solving process in the society through the innovation system. Accordingly, in this section, the structural components of the system (operational parts of the system or its actors) are identified. In Table 3, the most important actors of this problem are identified in three stages: those who do preventive and health actions before the disease, those who do therapeutic and operational actions during the disease, and those who do control and caring actions after the disease. These institutions and their actors can be separated into two levels, primary and secondary circles. The primary actors in the problem of cardiovascular diseases means the institutions and actors that deeply and directly affect the problem, and the secondary cycle means the institutions and actors that are at a greater distance from the operational core of the problem. Some institutions indirectly affect all these three levels and two cycles. For example, the Planning and Budget Organization,^1^ the Ministry of Agricultural Jihad,^2^ and the Ministry of Information and Communications Technology^3^ will have an undeniable role in allocating budgets and credits, cultivating healthy food and reviving and promoting the cultivation of medicinal plants, and forming the electronic medical file, respectively. Table 3 does not include all the institutions and actors related to the problem because an important part of the institutions and actors have a temporary presence and as they have important effects on the problem, this may defeat management and control policies for cardiovascular diseases. Therefore, for creating a system for the problem of cardiovascular diseases, most of the goals of these institutions and actors should be aligned.

**Table 3:** Levels of actors and main institutions related to the innovation system of the problem of cardiovascular diseases.

Preventive and Health actions (before the disease)		Therapeutic and operational actions (during the disease)		Control and caring actions (after the disease)	
Primary cycle	Secondary cycle	Primary cycle	Secondary cycle	Primary cycle	Secondary cycle
Deputy for public health Food and drug Organization Cardiovascular Diseases Working Group of the National Committee for the Prevention and Control of Non-Communicable Diseases of Iran	Parliament Health and Treatment Commission Planning and Budget Organization Ministry of Agricultural Jihad Deputy of Physical Education and Health of the Ministry of Education National media organization State Welfare Organization of Iran Deputy of public sports of the Ministry of Sports and Youth Department of Environment	Deputy for treatment Deputy for public health Emergency medical services Organization Food and drug Organization Cardiovascular Diseases Working Group of the National Committee for the Prevention and Control of Non-Communicable Diseases of Iran	Parliament Health and Treatment Commission Planning and Budget Organization Iranian Red Crescent Society Deputy of social affairs Iran Health Insurance Organization Social Security Organization Health Charities	Deputy for public health Deputy for treatment Deputy of social affairs	Food and drug Organization family institution

### 3.2 Analysis of the functions of the problem-oriented innovation system for cardiovascular diseases in Iran

In this section, the functions of the problem-oriented innovation system in cardiovascular diseases are examined in order to recognize the system inefficiencies through its weak functions. Weakness in functions can be caused by weak performance of structural components or actors or their absence in the relevant function. For this purpose, the functions of the innovation system must first be analyzed (Table 4) and the threats and weaknesses of each function must be addressed. Since each function of the system is realized based on its actors’ activities, each function is analyzed based on the actors’ performance. Based on this, in the following, the PIS functions of Iranian cardiovascular diseases (F_1-8_), related strengths (S_1-18_), and weaknesses (W_1-18_) are analyzed.

**Table 4:** The functions of problem-oriented innovation system (15).

Functions	Definitions
System guidance (F1)	The set of activities that lead to planning, legislation, allocation of resources, monitoring, and evaluation in the innovation system.
Technical and social knowledge creation (F2)	A set of activities that lead to the creation of knowledge with the aim of developing technology, investigating the effects of a problem on society, or providing a social, economic, and technological solution to solve a problem. This knowledge can be produced through different types of research, including basic, applied, developmental, etc.
Technical and social entrepreneurship (F3)	A set of activities in order to use technology in creating products/services, to eliminate or reduce the negative effects of the problem.
Knowledge dissemination, collaboration and synergy (F4)	The set of activities that lead to the dissemination of knowledge and cooperation between PIS subsystems and the components in each subsystem.
Standardization (F5)	Determining the acceptable level of risks to control the negative effects of the problem
Public awareness (F6)	A set of activities that lead to increasing public concern and knowledge about the problem and its solutions.
Resource mobilization (F7)	A set of activities to support the creation of knowledge by facilitating technical and social research, technology development, and standardization.
Market formation (F8)	A set of activities to create demand for technologies and products that reduce the negative effects of the problem.

#### 3.2.1 The function of system guidance

This function refers to the set of activities that lead to planning, regulation, resource allocation, monitoring, and evaluation in the innovation system. The constitution considers the need for healthcare services and medical care as a universal right (S1). Therefore, in order to achieve this universal right, various institutions with numerous actors have an effect on the problem. Although the Ministry of Health, and Medical Education and its subordinates are official institutions related to the problem, most of the decisions in the field of health are taken by groups that are outside the official cycle of the health system (W1) and have a strong impact on its structure (58); Therefore, policy makers outside the health sector should be aware of their effects in the health sector (59). The system guidance function of the system has had significant examples in the problem of cardiovascular diseases in Iran.

In this regard, the Ministry of Health and Medical Education is required to adopt strategies until 2025 to reduce the burden of cardiovascular diseases. In 2005, the Council of Ministers passed regulations on the advertisement of food products in national media, banning the distribution of carbonated soft drinks and removing its support policies, reducing trans fatty acids in edible oils and changing its quota to olive oil and special oil for frying, and promoting the cultivation of healthy oilseeds such as rapeseed and olives (S2)^4^. In 2021, in response to the shortage and unbalanced distribution of doctors, there was an agreement on the annual increase of 20% in the recruitment of medical students in less developed and deprived areas compared to the previous year for a period of 4 years (S3). However, maybe the most important attitude of this function in Iran’s health system can be seen in 2014, in the health system transformation plan. This plan was developed and performed to reduce the out-of-pocket payments of hospitalized patients (S4), increase the longevity of doctors in deprived areas, the reside of specialist doctors in hospitals (S5), improving the quality of examinations (S6), financial support for terminally ill patients and insurance coverage for the uninsured population (S7) (60). However, in the end, in addition to imposing high costs, it led the system toward treatment-oriented (W2) and its continuation could lead to the bankruptcy of the country’s insurance system (61). Therefore, policies resulting from the function of guiding the system in achieving goals should be reviewed regularly and periodically. The diversity and multiplicity of actors (W1), the interference and overlap of the duties of the health system with other executive bodies of the country, and as a result, the lack of transparency of the responsibilities and powers of individuals (W1) and the weakness of monitoring the correct implementation of policies (W3), are the most important threats that the system guidance function of the system is facing.

#### 3.2.2 The function of technical and social knowledge creation

**Figure 3:**
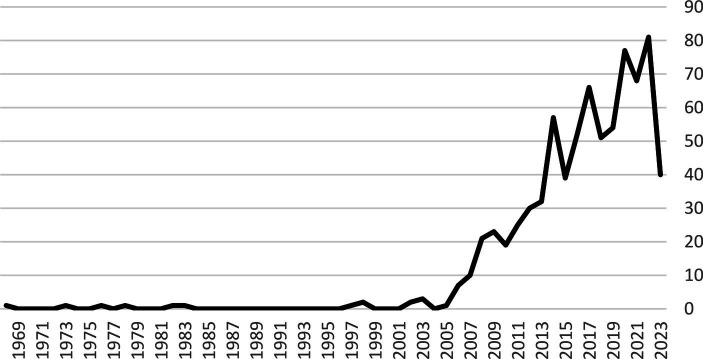
Scientific productions of Iranian authors in the field of cardiovascular diseases based on SCOPUS dataset until 2023.

This function deals with the creation of knowledge with the aim of developing technology to respond to the needs of society in order to solve problems. In Iran, since Razi and Pasteur institutes were established in 1919 and 1931 (S8), relatively appropriate measures have been taken to develop the technical knowledge of the pharmaceutical products relying on biotechnology knowledge (62). In relation to the knowledge creation in the field of health, Iran has made significant progress, so that the number of scientific articles in this field from 1990 to 2009 is 36.5 times that of 1980 to 1989 (S9) (63). In the problem of cardiovascular diseases, technical and social knowledge creation takes place at two levels: the creation of specialized and academic knowledge for the elite and the creation of general knowledge for members of society. The remarkable point is that the majority of elite knowledge is universal and there is no specific boundary in its use (S10). In this regard, the Google Scholar database has indexed more than 400,000 articles with the keyword “cardiovascular diseases” only in the period from 2019 to 2022 (S9). Also, the scientific productions of Iranian authors in the field of cardiovascular diseases, have increased since 2010 based on the SCOPUS dataset (S9). Its diagram is shown in Figure 3.

Finally, the lack of technology provision from universities in Iran’s biotechnology industry (W4) (64), and conducting studies that were not based on national needs or their results were not implemented (W4) (63), are the most important threats faced by the creation of technical and social knowledge.

#### 3.2.3 The function of technical and social entrepreneurship

This function refers to using knowledge and technology in order to eliminate or adjust the adverse effects of the problem. This function takes advantage of the technological results obtained from the knowledge dissemination function, and is influenced by the market formation function. The complicated relationships between actors (W1) and the institutional gap between medical equipment manufacturers and doctors and the lack of attention to innovative measures in treatment methods (W5) have caused the knowledge gained from the function of knowledge creation to be well created at the level of articles, but rarely being used in research and development (W4) so that this function is not basically formed. The health system’s scientific institutions and medical sciences universities in Iran have produced significant examples of laboratory or pharmaceutical products in a process based on research and development on a laboratory scale (S11), but these products need to be effective. They have been approved in clinical groups and after obtaining the relevant approvals, they should be given to patients so that the innovation can be commercialized by entrepreneurs (W6). Although drugs and basic medical equipment are exempted from economic sanctions, the direct and indirect effects of sanctions have restricted Iran’s banking system and, as a result, have created extensive restrictions on trade, production, insurance, and investment (W7) (65). On the other hand, sanctions make generic production difficult for domestic producers (W7) (66).

In general, sanctions are the most important threat to the function of technical and social entrepreneurship. In addition to this, the weaknesses of the market formation function have also limited the entrepreneurial opportunities in this function.

#### 3.2.4 The function of knowledge dissemination, collaboration, and synergy

This function explains the capability of the innovation system in the exchange of knowledge and technology between different actors of the system. In order to establish a health approach in all policies, the position of inter-sectoral cooperation (between the health sector and other sectors) in Iran’s health sector is at a favorable level in terms of explicit support in laws and documents and also the organizational structure (S12). This needs design and institutionalization of implementation mechanisms for increasing cooperation and building capacity (cultural and skill-based) for the stakeholders inside and outside the health sector (21). In the institutional and organizational dimension, apart from the Ministry of Health, seven institutions are responsible for more than half of the health share in the country (W1). Most of the activities of the cooperating institutions have been ignored and the successes have been attributed to the Ministry of Health and Medical Education (w8) (67). Also, in the individual dimension, studies show that from 2002 to 2011, three-authored articles have the highest number of articles and single-authored articles have the lowest number of articles in the field of cardiology (S13). There is also evidence that indicate Iranian researchers are more interested in internal collaborations (W9). In terms of collaboration indicators, scientific collaboration among authors in the cardiovascular field is at a favorable level and has been growing (S13) (68).

The threat in the organizational dimension is that most pharmaceutical companies are trying to carry out their innovative activities individually (69) and the cooperation between these companies is neglected (W9) (70). In the individual dimension, the most important threat to this function is “illusion of knowledge” with examples of self-treatment (W10) and “bio-technophobia” with examples such as lack of trust in the health system, avoidance of specialized treatments and vaccinations (W11). The dissemination of non-scientific materials in the field of health in a wide range through Internet & social media plays an important role in the disruption of this function. Meanwhile, the role of profiteers in weakening the health scientific knowledge should not be ignored (W10).

#### 3.2.5 The standardization function

The function of standardization means determining a minimum level of risks caused by the problem to control its negative effects. Although standardization through government requirements may inhibit innovation to some extent, it increases trust in high-risk products and attracts users’ trust and increases use by pioneers in the field and makes new products acceptable to a wider range of people (S14) (71). In Iran’s pharmaceutical industry, the pharmacopoeia standards and Good Manufacturing Practice are among the most important rules set by the Food and Drug Organization (S14) (72). Another important aspect of this function is determining the necessary criteria and standards in food preparation. The permissible amount of additives, salt and other substances in products will be explained through this function (S15). This function has a lot to do with the system guidance function because the necessary standards are converted into executive instructions through the system guidance function of the system. Additionally, the large number and lack of transparency of standards and measures (W12) (73) is one of the important threats to this function.

#### 3.2.6 The public awareness function

This function refers to the level of sensitivity and concern of society toward a public problem. Expanding public awareness of the signs and symptoms of cardiovascular diseases and necessary measures to control risk factors and early diagnosis of cardiovascular patients or people with high risk can reduce the incidence of complications and mortality of patients (S16) (14). Preventive behaviors of cardiovascular diseases should start from adolescence, when people’s understanding of the risk is low (35). Since this disease rootes in childhood, it is necessary to give people the necessary knowledge about cardiovascular diseases through proper education, so that they can prevent the occurrence of cardiovascular diseases and death caused by them by adopting proper health behavior. Therefore, the implementation of educational programs to adopt a healthy lifestyle in the early years of life is suggested (13). Teaching the healthy heart program is very effective in improving the level of awareness and attitude of students (S17) (22). Preparation of effective educational programs in the field of improving nutritional performance and physical activity of people, especially teachers, should be considered (74). It seems that with public education, we will see a reduction in the prevalence of risk factors and diseases (S16).

Health policy makers should design and compile extensive programs to change lifestyle at the society level, the main factors of which include increasing physical activity and improving nutrition (39). The low level of awareness and unfavorable performance of people in avoiding risk factors indicate predisposing factors for cardiovascular diseases (W10) (74). Low health literacy reduces people’s demand for health and medical facilities, and these people do not go to health and medical centers until the disease worsens (W10) (40). Some people also consider health recommendations to be ceremonial and do not take them seriously (W10).

The inadequacies of the health market, the great difference in knowledge between the providers and receivers of health services, and the intervention of health insurance require that the government use mechanisms to assure the people that it will provide high quality services that meet their needs (20). The most important threat to this function is satisfaction with environmental advertisements, hospital pamphlets and educational posters and ignoring the formal education of students through schools (W13).

#### 3.2.7 The function of resource mobilization

This function is a set of activities that provide the required resources for the operation of other functions. In the problem of cardiovascular diseases, this function has three aspects: “human resources provision,” “financial provision” and “equipment provision,” the most important of which is the provision of efficient human resources in the form of doctors and treatment staff in general. Although Iran has qualified doctors with high professional and specialized knowledge, it is not enough. There are 2,304 cardiologists, 214 subspecialists in cardiovascular surgery, 124 cardiac anesthesia fellowships, 66 vascular surgery subspecialists and 15 heart failure fellowships working in Iran (30). The unfair distribution of doctors with a high concentration in urban areas has a negative effect on the public health of the society (W14) (75). The emigration of elite medical staff (specialist doctors, general doctors, pharmacists and paramedics) has also increased and 79.8 percent of the participants in a research have had a moderate to high desire to emigration (76). Currently, there is a new wave of emigration in newly graduated doctors (32). The emigration rate of Iranian doctors during the covid-19 pandemic was facing a decrease due to global restrictions, but after the covid-19 pandemic, it has had an increasing rate and this is an important threat to the function of providing resources (W14).

In terms of financing, about 5.7% of the GDP is spent on healthcare costs (77). This cost is so high and harmful for the patients that is called “financial toxicity” in the literature (W15) (78).

Additionally, in terms of equipment, Iran is an importer of capital medical equipment in most cases (W16), but there is no comprehensive data bank about the distribution of these equipments in the country. This is while, for example, access to a CT-angio device causes a severe reduction in diagnostic angiography cases and as a result increases patient safety and reduces costs (34).

#### 3.2.8 The function of market formation

This function is a set of activities to create demand for technologies and products that reduce the negative effects of the problem. These activities can be pharmaceutical products of pharmaceutical companies and manufacturers of specialized equipment for heart patients inside the country. Although the number of research institutes in this field in Iran is satisfactory (S18), the number of biopharmaceutical companies is very limited (W17). A possible explanation for understanding the gap between research and drug production can be the inappropriate performance of the country in the field of commercialization (62). This is because most of those who are in the pharmaceutical industry are considered drug experts and are not familiar with business models (W18) (79). In general, international sanctions limit Iran’s pharmaceutical industry in three ways: The limitation of the industrial cooperation of pioneering multinational companies producing biological drugs with Iranian companies; The restriction of the sale of raw materials needed in this industry to Iranian companies; The prevention of the main equipment producers to sell their products to Iranian companies (W7) (80).

#### 3.2.9 Relationships between PIS functions

After explaining the functions in the previous sections, a total of 18 strengths (S) and 18 weaknesses (W) were identified in the functions of the Iran’s PIS of cardiovascular disease, which are listed in Tables 5, 6.

**Table 5:** The strengths and opportunities related to the eight functions of the innovation system based on the problem of cardiovascular diseases in Iran.

Strengths	Symbol	Relationships between functions
The universal right to health according to Article 29 of the Constitution	S1	F1, F6
Approving the laws for the management of advertising food products in national media, banning the distribution of carbonated soft drinks and eliminating its supporting policies, reducing trans fatty acids in edible oils, and promoting the cultivation of healthy oilseeds such as rapeseed and olives.	S2	F1
20% annual increase in the recruitment of medical students in less developed and disadvantaged areas compared to the previous year for 4 years	S3	F1, F7
Reduction of out-of-pocket payments for hospitalized patients compared to the past	S4	F1
Persistence of doctors in deprived areas and stay of specialist doctors in hospitals	S5	F1, F7
Improving the quality of examinations in the field of cardiology	S6	F1
Financial support for patients and insurance coverage for the uninsured population	S7	F1
Long-term activities of Razi and Pasteur Institute in creating technical knowledge	S8	F2, F4
The increasing growth of scientific articles in the field of health, especially in the field of cardiology	S9	F2, F4
Universality of elite knowledge in the field of cardiology	S10	F2, F4
Significant examples of laboratory or pharmaceutical products in Iran	S11	F2, F3, F4, F8
Desirable inter-sectoral cooperation (between the health sector and other sectors) in Iran in terms of explicit support in upstream laws and documents as well as organizational structure.	S12	F1, F2, F4
Scientific cooperation for writing papers in the field of cardiology	S13	F2, F4
Increasing trust in high-risk pharmaceutical cardiac products through standardization	S14	F3, F5, F8
Determining the necessary criteria and standards in food preparation and the permissible amount of additives, salt and other substances in products	S15	F5
Expanding general and public awareness of the signs and symptoms of cardiovascular diseases and necessary actions to control risk factors and early diagnosis of patients or people at high risk of cardiovascular diseases	S16	F6
Healthy heart program in schools	S17	F6
Acceptable number of research institutes in the field of cardiovascular diseases	S18	F2, F4

**Table 6:** Weaknesses and threats related to the eight functions of the innovation system based on the issue of cardiovascular diseases in Iran.

Weaknesses	Symbol	Relationships between functions
The diversity and multiplicity of actors inside and outside the health system, the interference and overlap of duties and, as a result, the lack of transparency of responsibilities and authorities	W1	F1
Treatment-oriented attitude	W2	F1
Weakness in monitoring the correct implementation of policies	W3	F1
Weak deployment of knowledge in research and development and lack of technology supply from universities in Iran’s biotechnology industry due to conducting research that are not based on national needs or their results are not implemented.	W4	F2, F3, F4, F8
Lack of attention to innovative actions in treatment methods	W5	F4, F8
Weakness in obtaining approvals related to the effectiveness of laboratory and pharmaceutical products in approved clinical groups	W6	F4, F8
Sanctions and restrictions on Iran’s banking system and, as a result, extensive restrictions on trade, production, insurance and investment.	W7	F4, F8
Ignoring the actions of cooperating institutions and attributing successes to the Ministry of Health and Medical Education itself	W8	F4
Researchers’ desire for internal collaborations in paper writing and companies’ desire to carry out individual innovative activities	W9	F4
Low level of awareness, publication of non-scientific materials in the field of health and “illusion of knowledge” with examples of self-treatment, and not taking health recommendations seriously in the individual dimension	W10	F2, F4, F6
“Biotechnophobia” with examples such as not trusting new drugs and avoiding specialized treatments	W11	F4, F8
A large number and lack of transparency of standards and measures	W12	F5
Satisfaction with environmental advertisements, hospital pamphlets and educational posters and ignoring the official education of students through the Ministry of Education	W13	F6
Potential shortage of specialist doctors, increasing emigration and uneven distribution of available doctors	W14	F7
Imposing back-breaking costs on patients	W15	F1
Supplying medical equipment only through imports	W16	F7
Limited number of biopharmaceutical companies	W17	F2, F3, F4, F8
Lack of familiarity of pharmacists with business models	W16	F3, F8

Although according to Tables 5, 6, each function of the problem-oriented innovation system separately has strengths and weaknesses, they simultaneously are continuous, dynamic, mixed and sometimes conflicting with each other, which is shown in the last column of the tables. For example, strengthening the function of public awareness in cardiovascular diseases can change the function of system guidance through public will and even require it to legislate and change the allocation of resources depending on the level of public awareness. Public awareness can increase the demand for products and drugs that are effective in controlling cardiovascular diseases and lead to the market formation, develop the technology, and finally, strengthen the commercialization of innovations. This also requires resources and legislation. A part of the input of research centers are problems that have been highlighted by public concerns and awareness and forms the basis of future researches, and consequently, lead to the creation of technical and social knowledge. Public awareness in the form of mass media and scientific meetings will result in the dissemination of scientific findings and synergies. Public awareness, especially among the scientific community, encourages people to analyze the problem to find possible solutions for the function of technical and social knowledge. In the problem of the challenges of cardiovascular diseases, the function of public awareness has an urgent action. Although most of its direct or indirect effects are long-term, it prevents the system to get weaker. It is expected that the strengthening of this function will manage the challenges in the society-oriented layer, in particular, “insufficient education,” “unhealthy nutrition” and “unhealthy lifestyle and cultural weakness.”

The function of technical and social knowledge creation provides data for policy makers and legislators, and this function strengthens system guidance and standardization. The strong performance of the technical and social knowledge creation function is dependent on directing knowledge in the right direction through optimal policy making and allocating sufficient resources to support the policy. The scientific results of the function of technical and social knowledge creation will cyclically modernize the system and regularly review and modify the demand and laws, and finally, it will promote public awareness. By strengthening this function, it is expected to manage the challenges of “legislative and political vacuum,” “insufficient education,” “unhealthy nutrition,” “unhealthy lifestyle and cultural weakness” and an answer to the challenge of “uncontrollable and hard to adjust factors” in the future.

In the function of system guidance, the establishment of regulations can show its effectiveness in a short period of time, but the laws and regulations that have weak cultural support will not last, so the change in behavior that is caused by awareness can be effective in promoting other functions of the system, even if its results appear in the long term. Therefore, it is expected that strengthening this function can manage the challenges of “legislative and political vacuum,” “insufficient training,” “shortage and unbalanced distribution of medical personnel,” “unhealthy lifestyle and cultural weakness,” “financing,” and “high cost of diagnosis and treatment.” If this function is combined with the function of public awareness, it will be more effective.

The competitiveness of companies active in national and especially international markets depends on the extent to which these companies comply with the relevant standards. Therefore, by strengthening the function of standardization, it is expected that the function of market formation will also be strengthened and the challenge of “legislative and pilicy gap” will be managed. Strengthening the function of technical and social entrepreneurship, effects the function of market formation in a two-way relationship, and both functions will be effective in managing the “financing” challenge.

Strengthening each function not only helps to solve the problem, but directly or through another function, it will facilitate and strengthen the system. Similarly, incorrect operation can weaken and even disrupt the performance of a problem solving system, so it is necessary to intervene in system reform and remove obstacles in a cycle of reorganizing functions.

## 4 Discussion and policy recommendations

**Figure 4:**
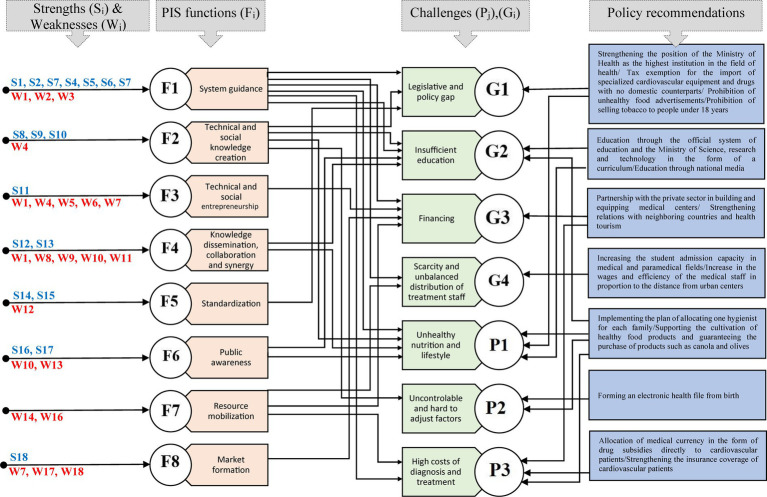
Policy recommendations, relationships between functions, strengths, weaknesses and challenges related to the problem of cardiovascular disease.

After examining the strengths and weaknesses affecting the functions of the cardiovascular disease system in Iran, it is clear that for the systematic management of this challenge, it is necessary to control and eliminate the weaknesses, and strengthen the strengths and opportunities in supply side, demand side and on the supply and demand exchange side. Therefore, policy recommendations should be provided for strengthening the strengths and controlling or eliminating the weaknesses so that the functions of the system are improved and then the grand challenge of cardiovascular diseases is managed. These relationships are shown in Figure 4.

In the problem of cardiovascular diseases in Iran, the position of the Ministry of Health as the highest institution related to the issue in the country should be established and the decision-making position of other actors and institutions outside the system should be defined. In this regard, it is necessary to formulate and approve the protective and deterrent laws of the health system (including challenges related to cardiovascular diseases) in the parliament at the two levels of state-oriented and society-oriented. At the state level, it is necessary to exempt tax and facilitate the import of medical equipment and cardiovascular drugs, especially equipment that does not have domestic equivalents. Additionally, it is necessary for the Ministry of Health and Medical Education to create an electronic health file for all people in the country from birth in cooperation with the Ministry of Communications and Information Technology, and monitor its status every six-month. This helps Ministry of Health and medical education to monitor the data related to the number of cardiovascular patients, the number of people with acute medical conditions, the amount of consumption and the type of drugs prescribed, the country’s drug needs (both in production and import), in different time frames and provided to health decision-makers under the “Comprehensive System of the Countries’ Drug Needs.”

Supporting the cultivation of healthy food through specialized consultations of experts in the field of agriculture, and guaranteeing the purchase of healthy food products by the state-oriented layer should also be considered.

It is necessary to support the deterrent policies approved by the judicial system of the country. Policies such as banning the use of the national media to advertise harmful and dangerous foods, banning the sale of tobacco to people under 18 years of age, or charging a health tax for tobacco and products such as carbonated drinks and sugary substances should be taken seriously. We can expect the implementation of deterrence policies only when the punishment for violating them is clear and proportionate.

In order to provide human resources, it is necessary to increase the admission capacity in medical and paramedical universities by 25% every year. In order to maintain human resources in less developed areas, the state-oriented layer should consider the increase of salaries and wages in relation to the distance of the resident doctors from the capital and the centers of the provinces. In order to provide financial resources for projects in the field of health and treatment, the government sector can set up specialized and sub-specialized centers in the field of heart and blood vessels in underprivileged areas that lack basic equipment, through joint investment with individuals and the private sector, up to 75% participation, to encourage investment in the field of health and treatment. The participation shares in areas that face a severe shortage of specialized centers can be 75% for the government and 25% for the private sector. In other areas, the percentage of participation can change according to the needs.

Although one of the weaknesses of the cardiovascular disease system is the diversity and multiplicity of actors inside and outside the health system, the diversity and multiplicity of actors is inevitable due to the magnitude of the problem and this can be an opportunity for cooperating with other institutions. One of the most important institutions in the field of formal education is the Ministry of Education and the Ministry of Science, Research and Technology centered on their students. In order to strengthen the function of public awareness and education, it is recommended to include educational and academic units related to the problem of cardiovascular diseases in the schools and university’s curriculum.

The Ministry of Health, can play an effective role in strengthening the function of public awareness by holding face-to-face or virtual courses through simple and basic health and care training such as cardiac resuscitation training, control and screening of blood pressure and blood sugar, and providing health advice to one member of each family. This important action can be started from rural and less developed areas.

Effective communication with regional countries, especially neighbors, can reduce the effects of sanctions on the country’s health system to an acceptable extent. Transnational communication strengthens the dissemination of knowledge and scientific cooperation between researchers and also improves the level of standards in line with commercial relations. Considering Iran’s strategic position in the region, attracting health tourists is also an important step in strengthening the function of regional market formation and strengthening the function of technical and social entrepreneurship.

## 5 Conclusion

Most of the problems for health policy makers are multi-dimensional and malignant, and even some of them have solutions beyond the borders of the health system. Cardiovascular diseases, as one of the non-communicable diseases, are one of the most important challenges for the health system of any country. In essence, such problems not only affect the health system, but also the economic, social and cultural systems, and even the political system of a country. Different governments have different policies in the face of cardiovascular diseases. A group of policies are focused on controlling the costs of cardiovascular diseases. Some of them are health or cultural preventive actions, or they simply have an awareness aspect. In some cases, policies have a legislative and regulatory aspect, and some others are deeply affected by the political developments of a country. This has caused countries to face a complex phenomenon and interactions of many actors in the face of cardiovascular diseases. Such problems have created more concerns especially for developing countries such as Iran, which in some cases have weak innovative capacities and institutional arrangements. Therefore, solving their problems requires systematic and innovative solutions. In this regard, the problem-oriented innovation system (PIS) approach was used in this paper to solve the grand challenge of cardiovascular diseases.

PIS is an analytical tool based on a systematic approach that provides the possibility of functional-structural analysis at the same time and thus, helps to extract policy implications in the management of grand challenges such as cardiovascular diseases. This is a new approach that has not been used in Iran and considers the issue beyond the geographical borders.

One of the most important differences between the problems of the health system, such as cardiovascular diseases, and other problems in other areas is that solving this problem is urgent and parts of it cannot be solved by trial and error and valuate its consequences economically. Any wrong policy can have irreparable consequences. Therefore, solving grand challenges such as cardiovascular diseases require interdepartmental and multifaceted technological and social solutions.

In this study, the challenges of cardiovascular diseases were categorized in two levels: state-oriented and society-oriented. Paying attention to just health and treatment or economic aspects does not lead to a sustainable solution to such problems; The problem-oriented innovation system approach has the same characteristic. This approach tries to analyze the problem from health and treatment, technical, economic, cultural and social aspects. In other words, this system pays attention to the application of innovation in solving social problems and considers the boundary of the system wider than the health system, so it is likely to provide a sustainable solution in solving the problem. This approach presented eight functions for solving the problem, which provides a tool for the policy makers in the problem of cardiovascular diseases to investigate the problem in a systematic and comprehensive way, identify the main bottlenecks that has led to the emergence of the problem, and fix them. This framework has a mixed approach in policymaking and considers both bottom-up and top-down approaches.

This research has had some limitations. One of the most important limitations of this study was the extraction of accurate data related to Iranian cardiovascular patients. In this study, the problem-oriented innovation system was used in the management of the grand challenge of cardiovascular diseases in Iran, but this approach, beyond the geographical border, can be used for other countries as well. The emphasis of this study is on cardiovascular diseases as one of the non-communicable diseases, so it is recommended that researchers use this innovative systematic approach to the control and management of infectious diseases in future researches. This is the same for similar cases that have an increasing growth rate or that require everyone’s determination at the national and international levels. Also, the following studies are suggested for future research based on the PIS framework:

- Conducting a comprehensive analysis of Iran’s healthcare infrastructure, including urban–rural disparities.
- Assessing the economic implications of interventions, considering healthcare financing, and socioeconomic disparities.
- Surveillance of cardiovascular disease epidemiology to identify high-risk populations and geographic areas.

## Data availability statement

The original contributions presented in the study are included in the article/supplementary material, further inquiries can be directed to the corresponding author.

## Author contributions

SN: Writing – original draft, Writing – review & editing. JA: Writing – original draft, Writing – review & editing. GS: Writing – review & editing. SG: Writing – review & editing.

## Footnotes

^1^https://www.mporg.ir/

^2^https://maj.ir/

^3^https://www.ict.gov.ir/

^4^https://rc.majlis.ir/fa/law/show/127600
